# User Profiling to Enhance Clinical Assessment and Human–Robot Interaction: A Feasibility Study

**DOI:** 10.1007/s12369-022-00901-1

**Published:** 2022-07-08

**Authors:** Laura Fiorini, Luigi Coviello, Alessandra Sorrentino, Daniele Sancarlo, Filomena Ciccone, Grazia D’Onofrio, Gianmaria Mancioppi, Erika Rovini, Filippo Cavallo

**Affiliations:** 1 Department of Industrial Engineering, University of Florence, Florence, Italy; 2 The BioRobotics Institute, Scuola Superiore Sant’Anna, Pontedera (Pisa), Italy; 3 Department of Excellence in Robotics & AI, Scuola Superiore Sant’Anna, Pisa, Italy; 4 The Complex Unit of Geriatrics, Department of Medical Sciences, Fondazione “Casa Sollievo della Sofferenza” – IRCCS, San Giovanni Rotondo, Foggia, Italy; 5 Clinical Psychology Service, Health Department, Fondazione IRCCS Casa Sollievo Della Sofferenza, San Giovanni Rotondo, Foggia, Italy

**Keywords:** User profiling, Social assistive robot, Multimodal sensors, Robot behavioral model

## Abstract

Socially Assistive Robots (SARs) are designed to support us in our daily life as a companion, and assistance but also to support the caregivers’ work. SARs should show personalized and human-like behavior to improve their acceptance and, consequently, their use. Additionally, they should be trustworthy by caregivers and professionals to be used as support for their work (e.g. objective assessment, decision support tools). In this context the aim of the paper is dual. Firstly, this paper aims to present and discuss the robot behavioral model based on sensing, perception, decision support, and interaction modules. The novel idea behind the proposed model is to extract and use the same multimodal features set for two purposes: (i) to profile the user, so to be used by the caregiver as a decision support tool for the assessment and monitoring of the patient; (ii) to fine-tune the human–robot interaction if they can be correlated to the social cues. Secondly, this paper aims to test in a real environment the proposed model using a SAR robot, namely ASTRO. Particularly, it measures the body posture, the gait cycle, and the handgrip strength during the walking support task. Those collected data were analyzed to assess the clinical profile and to fine-tune the physical interaction. Ten older people (65.2 ± 15.6 years) were enrolled for this study and were asked to walk with ASTRO at their normal speed for 10 m. The obtained results underline a good estimation (*p* < 0.05) of gait parameters, handgrip strength, and angular excursion of the torso with respect to most used instruments. Additionally, the sensory outputs were combined in the perceptual model to profile the user using non-classical and unsupervised techniques for dimensionality reduction namely T-distributed Stochastic Neighbor Embedding (t-SNE) and non-classic multidimensional scaling (nMDS). Indeed, these methods can group the participants according to their residual walking abilities.

## Introduction

During the last decade, increasing research interest in Socially Assistive Robotics (SAR) has brought researchers to develop intelligent robotic solutions that assist users through advanced social interaction capabilities. The objective of SAR systems is to provide continuative support and assistance with appropriate and contextualized emotional, cognitive, and social cues through the creation of close and effective interaction. Such systems were used to provide support and assistance to a wide range of users, including frail older adults in domiciliary and hospital settings thus improving their quality of life [1, 2]. Indeed, one of the required distinctive traits of SARs relies on their capabilities to perceive, learn, and recognize models of the other agents it is interacting with [3].

Remarkable results have been achieved with SARs in realistic scenarios involving real users (see e.g., [4, 5], also concerning the assistance and the monitoring of impaired and frail people (see, e.g., [6–8]. However, a crucial requirement for effective SAR systems is the capability of dealing with a high variety of situations and contextualized interactions according to different living contexts and cultural habits of assisted people showing a high degree of adaptability [9]. In this sense, the Human–Robot Interaction (HRI) field has become crucial, and it is now compelling better understand how humans perceive, interact with, or accept these machines in social contexts. Consequently, there is a growing interest in the development of models that can enhance the interaction between humans and robots [10].

According to the literature [10, 11], the research efforts aim to develop innovative and adaptable behavioral models of robotic systems based on brain-inspired Artificial Intelligence (AI) cognitive architectures. Indeed, a substantial part of the research work in this area is addressing fundamental scientific problems in cognitive HRI and revolves around four main themes:

- the study of psychology, social cognition, and neuroscience models of human–human interaction (HHI);
- the development of sensing and perceptual abilities of robots to detect emotion, action, and other social cues, including real-world noisy data based on AI techniques;
- the development of bio-inspired social behaviors for a robot that continuously learns from humans and adapts to new social challenges showing human traits;
- the ethical, social, and legal implications of interaction and data management to consider to be compliant with privacy aspects.

Despite the crucial research potential of such topics, gaps from a scientific perspective are still present. It is needed to have more discussions and developments, and extensive testing in the field, for improving robot capabilities and clinically validating solutions for healthcare applications [12]. Indeed, the key challenge in this field is to provide robots with cognitive and affective capabilities, developing architectures that let them establish empathetic relationships with users. This requires an enormous effort in the fields of engineering and AI and covers areas such as face and emotion recognition, action and intention, prediction, speech processing, and many others.

Sensing and perception are two key robot capabilities to deeply investigate for deploying a personalized and contextualized HRI. When a robot interacts or, in general, coexists with humans, it should sense/perceive and understand the contexts and the humans. Human interaction is inherently multimodal: while interacting, we are continually producing and interpreting a rich mixture of data. In effect, in human–human interaction, the listener (i.e. the observer) automatically assesses the emotional and engagement state of the speaker (i.e. the actor). This human inherent ability in a social context is the effect of specific processes happening at the level of the brain, as described by the Theory of Mind [13]. To mimic this capability, SARs should be able to detect and recognize some bio-inspired communications signals, referred to as social cues, which could be “*descriptors of the behavioral state of the user during the interaction*”, namely: posture and body movements, facial expression, head orientation, verbal message, emotion, voice quality, gesture, vocalization [14]. Consequently, information on the hand movement, the direction of gaze as well as the relative position of body joints can be analyzed to enhance the perception of the user’s engagement during the interaction [15].

On the other side, clinical evidence showed that these social cues could give a spot, or it could be linked with physical, psychological, and cognitive impairments that could affect frail older persons. Alteration of muscle strength and walking velocity characterize sarcopenia which is a major contributor to the risk of physical frailty [16]. Additionally, the body postures (i.e. body orientation and interpersonal distance) could be expressions and symptoms of depression [17], indeed for humans, maintaining a standing posture is important for spatial recognition, positive physiological effects, and personal dignity.

In this context, the aim of this paper is dual:

- Firstly, we propose a robot model that aims to analyze the collected data extracting the cues that can be used as inputs for i) *Health Contextual Reasoning module* that leads toward a decision support tool for the clinicians by making data available thus to correlate them with symptoms (and diseases); and ii) *Interaction and Intervention Reasoning module* that orchestrates the interaction ability of the robot using AI algorithms to tailor the interaction with the users.
- Secondly, we aim to test the model in a feasibility study in a real environment. Particularly, we focused on the sensing and the perception modules implemented in a social robot, namely ASTRO. ASTRO robot [18] was designed to support the indoor walking task by providing personalized physical support thanks to the embedded sensors (i.e. laser and 3d camera on its back and 16 force sensors embedded on its handle). Particularly, in this paper, the cues related to the sarcopenia (i.e., handgrip strength, body posture, and gait parameters) were acquired and stored from those sensors during the walking assistance task with ASTRO and analyzed to profile the user. These aggregated outcomes could be used, at the same time, by the clinicians to evaluate the performance and to monitor the progress of the disease, and by the robot’s model to shape the physical interaction during the walking assistance task.

The remainder of the paper is organized as follows, Sect. “The Robot Model” introduces the robot model and details the research questions. Section “Related Work” presents an overview of the literature concerning the use of robots in the clinical setting and the commonly used sensors to extract the desired parameters thus contextualizing the sensors selection. Section “Sensing Module: Data Extraction” details the methodology such as the experimental setting and the data analyses conducted. Sections “Results” and “Discussion” present and discuss the results respectively. Finally, Section “Conclusion” concludes the work.

## The Robot Model

**Fig. 1:**
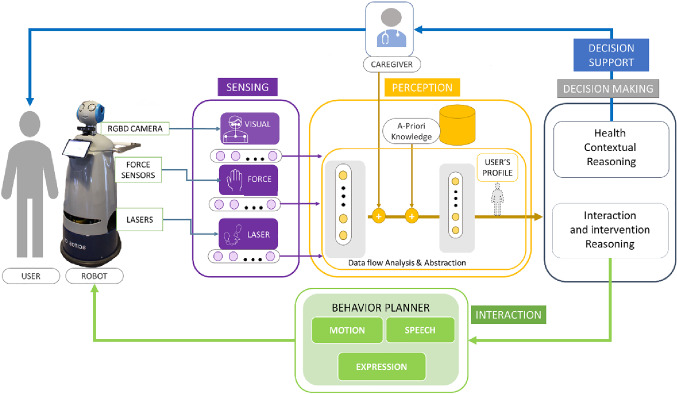
The proposed robot model includes (i) the sensing module (purple box) that collects multimodal data from the robot; (ii) the perception module (the yellow box) that processes and combines the data for the decision-making module (grey box) providing the decision support tool for the caregiver (blue box), and for the interaction module (green box)

Research in modeling robot behavior aims at endowing a SAR with three key stages that are usually performed during the HRI: the *sensing*, the *perception,* and the *interaction* (i.e. the *acting* of the robot) modules. At the same time, the SAR behavioral model should rely on the correct knowledge of (i) the needs and the profile of the assisted person; (ii) the context and its dynamic changes; and (iii) the abilities of the robotic platform, thus to adapt the robot *action* [3]. Research in AI has significantly contributed to different levels in the realization of the mentioned stages, obtaining also significant results in designing flexible and adaptable solutions [2]. In this context, we propose an architecture (Fig. 1) based on four main modules (i.e. sensing, perception, decision support, and interaction) and inspired by the ones described in [7, 9, 19].

Within the sensing module, the multimodal data collected by the platform are recorded and analyzed to extract features that characterize the selected social cues. The elaborated information is the input of the perception module.

The perception module fuses these features at different levels to perform autonomous user profiling. It could rely on machine learning algorithms (e.g. supervised learning, deep learning, reinforcement learning) that aggregate data and abstract information from the sensing module, data from clinical records (i.e. a priori knowledge) such as from the context to define the *user perception profile* (see yellow box in Fig. 1). User profiling is necessary to update robot knowledge about the (current) state of an assisted person (i.e. physical, cognitive, and social profiles) as well as implementing an effective interaction strategy and for providing correct information for the clinical assessment. Indeed, the robot model relies on the outcomes of the perception module to infer and plan the actions. As in [7], the proposed model has a professional-in-the-loop that could adjust the perception of the user profile providing the robot with some elements to tailor the process of the perception module.

The outcome of user profiling goes into the *decision-making module*, which explores the duality of the information. The rationale behind this is that the same cues could lead to different uses if analyzed in different contexts. As previously anticipated, the cues-related features could bring information on the quality of the interaction (i.e. engagement or emotion of the user), but also on potential alteration of the cognitive and physical status. In this sense, the *Health Contextual Reasoning* module elaborates the information for being used from the professional-in-the-loop; indeed, the professional caregiver could use the provided data as a support for the assessment, for the monitoring stages of a certain pathology, or for identifying anomalies that could be correlated with early sign of a disease. Alternatively, if the information about the user profile is used by the *Interaction and Intervention Reasoning module*, the robot could use this information as input for the *interaction* stage. It aims to adapt the interaction between the robot and the human beings according to their preferences/needs and the (current) status detected by the *perception module*. Indeed, it includes the behavior planner module that will use the information on the user profile to adapt the HRI in terms of motion, speech, and expressions. For instance, in the case of walking assistance, if the user perception module identifies an older person with slow gait velocity and incorrect body posture, the interaction module could adapt the behavior of the robot’s motion, by setting the appropriate angular and linear velocity of the motors.

Table 1 reports a list of features that describe social cues used by humans in communication. This table underlines the duality of their use in the context of HRI and in the field of clinical assessment. It is evident the importance of endowing the robotic platform with a multimodal sensing module to capture different cues. It also highlights the efficacy of an accurate perception module that can extract and organize the information in the best way to improve the HRI and provide support for the professionals.

**Table 1:** The same features could be used to estimate the HRI and to assess the clinical status of the user

Cues	Features	HRI relationship	Clinical assessment relationship
Body posture & movement	Body orientation	Engagement [59]	Depression [17], Apathy
	Interpersonal distance	Engagement [60]	Age [61], Psychopathy [62]
	Arm movement	Engagement [63] & Emotion [64]	Hints regarding the axis of apathy-agitation
	Gestures	Engagement & Emotion [7]	Apathy [65], Parkinson [66]
	Gait parameters	Engagement & Emotion [67]	Cognitive decline [68], Dementia [69], Parkinson [57], Sarcopenia [16]
Emotion	Facial expression	Emotion [70]	Apathy [71], Parkinson [72]
	Heart rate fluctuation	Emotion [73, 74]	Stress [75, 76]
Head orientation	Eye gazing	Engagement [77]	Attentional fluctuation [78]
Muscle strength	Hand grip strength	Physical HRI [79]	Frailty [16]
Voice quality	Tempo	Emotion [80]	Neurodegenerative diseases [81, 82]
	Energy		
	Pitch		
Verbal message	Repetitions	Engagement & Emotional [14]	Hints on mental flexibility and planning (repetitions), signs related to lexical problems (incomplete words) and hesitations (silence)
	Incomplete words		
	Amount of silence		

In this paper, we focused on testing the sensing and the perception modules of the ASTRO robot pointing to the use of data for the *Health Contextual Reasoning module*. For what concerns the use of data for the *Interaction and Intervention reasoning module* to adapt the HRI using AI we presented our results in [20]. The perception of the user state is established using ASTRO embedded sensors: the upper body posture was estimated with the RGB-D camera,while handgrip strength was measured by force sensors installed on the robot handle, and gait parameters were extracted from laser data installed on ASTRO’s back. Then, all the data were fused at the feature level to assess the status of the user. Summarizing, the research questions (RQs) of this paper are:

1. RQ I: to analyze the visual, force, and laser data to extract from ASTRO robot the parameters connected to body posture, handgrip strength, and gait respectively to investigate the goodness of the perception module.

We expect to analyze the acquired data and to extract the parameters related to the body posture, the gait, and the handgrip strength since these are linked with the evaluation and the monitoring of sarcopenia. We expect to have comparable measurements obtained with ASTRO robot and with wearable devices used traditionally (i.e. IMUs, hand dynamometer). We expect to not find significant differences between the two groups of extracted features. Particularly, the data related to the gait extracted from the ASTRO embedded laser will be compared with the features extracted from IMUs signals placed on the feet, since inertial sensors are widely used to measure the gait. As for the body posture, the body inclination measured with the ASTRO camera was compared with the inclination measured with the IMU placed on the sternum. Finally, we will investigate whether the handgrip strength measured with the ASTRO handle is not different (*p* > 0.05) from the ones measured with the dynamometer. These are all fundamental steps to be verified to confirm the goodness of the user profiling process and before using ASTRO robot as a tool for the decision support system.

2. RQ II: to investigate if the output of the sensing modules can be combined in the perception module to characterize the user profile during the walking task thus providing input for the decision support module.

Since the main goal of this paper is to present and discuss the user’s profile perception, as the outcome of the perception module, we expect to see that the combination of all the features extracted from ASTRO collected data will lead to a correct perception of the user profile thus to be used as the input of the decision support tool. Indeed, in this paper, we will analyze the data to verify if the features could be combined to describe the motor performances according to the user's residual physical abilities (i.e. user profile). Particularly, the features will be combined in a bi-dimensional space, and we will observe if users with a similar profile will be visualized at closer points.

## Related Work

As depicted in Fig. 1, this work represents the first step toward a new paradigm of socially assistive robotics since it relies on the duality of sensors data, which could be used by the robot both to adapt its behavior during the interaction and to provide feedback on the clinical user profile to the professional caregivers. Currently, the robots in a hospital setting are generally used for logistic services, such as transportation tasks, moving goods such as medical equipment [21], food and garbage delivery tasks [22], and saving human resources optimizing also task distribution and scheduling [23, 24] to improve the hospital services. However, these applications are not directly related to the clinical aspects in terms of diagnosis and monitoring neither to the social aspect of interacting with patients.

Important aspects to be investigated to enable the management, monitoring, and assessment of patient care in hospitals by SAR, is the trust in robotic decision support among nurses and doctors [25, 26] and the expectation toward robots [27]. Even if several issues arouse about the actual use of robots in clinical practice (such as economic feasibility, acceptance among clinical staff, acceptance among patients) [28, 29], the results obtained by Gombolay et al. [30] sustained that the robot provided high-quality recommendations for delivery tasks, with a compliance rate of 90% respect to the clinical experts. Additionally, D’Onofrio et al. [6] investigate the use of SAR in supporting the work of professionals in administering the comprehensive geriatric assessment that includes also physical and cognitive tests (e.g. sarcopenia, mini-mental state examination, Tinetti balance assessment tool). In this sense, having a system (or a network of systems) that could support and provide support during the administration of such tests could improve the assessment process.

It is evident that over the last years, researchers and clinicians started to investigate several technological solutions that could measure/analyze the output of such tests quantitatively. But which are the most used sensors? Above all, they were mainly focused on the analysis of body movements, fine gestures, or activities since their fluctuations can be correlated with neurodegenerative diseases (Table 1). In this sense, wearable sensors seem the most promising ones [31]. As described in [32, 33], the inertial data collected by the wearable sensors allows the extraction of the walking parameters in time and frequency domains [33, 34]. Similarly, smartphones were also used to measure walking parameters [35–37], thanks to the Global Positioning System (GPS) data and embedded IMUs. However, both these solutions could be cumbersome to be used by frail and older persons. Indeed, older people would not have any external sensors because sometimes they forgot to wear them.

Less invasive solutions are proposed by pressure-sensitive walkway systems (e.g. GAITRite [38], GEAR [39]) and the usage of optical motion capture systems, such as Vicon Motion System (Vicon Motion System Ltd, UK) or OptiTrack (OptiTrack, Corvallis, OR). While the former technology analyses the gait by quantifying the pressure patterns under a foot [40], the latter solution is composed of multiple cameras and 3D markers that need to be attached to the user’s body. Both types of technology are characterized by high fidelity and accuracy in movement reconstruction, but it is still a bit invasive, low portable (e.g. they cannot be used outside the laboratory settings [41]), and too expensive to be exploited in the evaluation of health conditions and during daily activities.

Due to this limitation, RGB and RGBD cameras are becoming promising alternatives. Indeed, they provide a marker-less and portable solution for the estimation of biomechanical gait features [41] and body posture [42]. These contactless sensors could be easily installed on a social robot that could use the inputs to provide feedback on the user profile during the interaction thus adapting its behavior and supporting the professionals ‘work.

Most of the RGB-D cameras (e.g. Microsoft Kinect) incorporate proprietary software for detecting the joints’ coordinates of the user body, which allows the estimation of biomechanical gait features of the lower [43, 44] and upper limbs [45–48]. Similar results have been obtained by the RGB cameras, due to the emergence of two popular machine learning models which allow for real-time human pose estimation: OpenPose [42] and PoseNet [49]. Both tools allow the extraction of 2D keypoints that could be used to perform gait analysis, as described in [41, 50, 51]. Authors in [52] used it to compute the distances of the joints from a reference point, manually selected on the first RGB frame. Also, Gu et al. [50] used OpenPose framework in conjunction with the GrabCut tool [53] to detect the foot position in the image. Both works focused on lower limb parameters estimation for gait analysis from a fixed camera. One common design choice in the mentioned works is the stable position of the camera. Namely, in most of the works the camera is fixed in one room, usually attached to the ceiling or at least 2 m distant from the user. One of the novelties of our work relies on the configuration of contactless sensors. Indeed, the camera used for the gait and body posture analysis is mounted over a moving robot. It allows the continuous monitoring of the elderly activity with no constraints on the sensor’s location. Furthermore, in this work, we aim to enrich the clinical parameters extracted with the wearable and vision sensors by including the gait parameters that can be assessed by the laser sensor mounted on the robotics platform.

## Material and Methods

### System Description

**Fig. 2:**
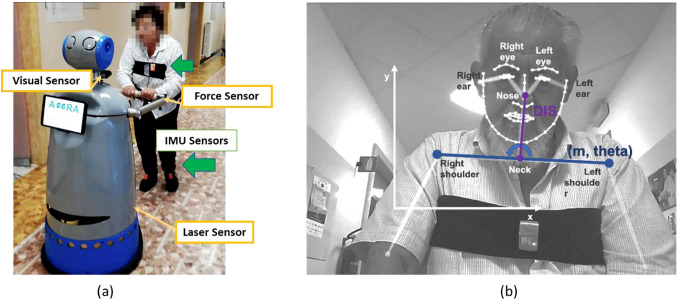
**a** A subject during the 10 m walk with the ASTRO robot. The yellow label indicates the sensors embedded on the robot whereas the green label and the green arrows indicated the wearable IMUs sensors. **b** OpenPose frameworks, the used joints are indicated such as the parameters extracted from the camera

ASTRO has been designed to assist an older person with mobility needs as refined under ACCRA project [54] (Fig. 2a). ASTRO is based on the SCITOS G5 robotic platform (Metralabs GmbH, Germany). SCITOS G5 is also equipped with a bumper and a couple of emergency-stop buttons that can stop the motor, if required, guaranteeing the safety of the users. On the front, the platform is improved with an additional laser (Laser Sick 300) enabling the perception of the surrounding environment and promoting autonomous safe navigation into the environment.

On the back, ASTRO is equipped with a second 2-dimensional laser sensor for area scanning (Hokuyo URG-04LX-UG01). The light source of the sensor is a pulsed light laser diode (infrared) of wavelength 785 nm with laser class 1 safety. The scan area is 240° semicircle with an angular resolution of 0.36º and a maximum scan distance of 4000 mm. Scans are performed at a frequency of 10 Hz. Additionally, an Astra ORBECC camera is mounted on the back of ASTRO to observe the upper part of the user during the walking task. Namely, it records RGB data with a resolution of 1280 × 720@30fps and depth images with resolutions 640 × 480@30fps up to 8 m.

About Human–Machine-Interface two touch screens for direct access to the GUIs were mounted on the front and the back sides of the robot.

On the back, ASTRO is equipped with a smart handle to support personal mobility. 16 force sensors (Flexiforce, Tekscan, USA) were mounted in correspondence of the right and the left hands to improve the physical HRI during the mobility services [55]. ASTRO has *a smart drive mode* that allows the users to use his/her handgrip strength to drive the robot during the walking support service. Thanks to this module, the data acquired from 8 force sensors embedded on ASTRO handle were combined in real-time using machine learning technique (i.e. decision Tree) to adapt the linear and angular velocities of the robot to the normalized force applied. As result, the robot could adjust the “drive mode” of ASTRO by changing the applied handgrip strength [20].

In this paper, three inertial sensors (9-axis) (XSens, Netherlands) were used to verify RQ I. These sensors acquire data at a sampling frequency of 100 Hz. Additionally, CAMRY hand dynamometer was used for the measurement of the grip strength as in traditional clinical practice. These sensors, external to the robot, have been used just in the experimental setting to verify the perceptive capabilities of the robot. They would not be necessary, therefore, in the standard use of the robot.

### Participants

A total of 10 subjects, 8 men and 2 women (65.2 ± 15.6 years) were enrolled among patients at the research hospital “Fondazione Casa Sollievo della Sofferenza” located in San Giovanni Rotondo (Foggia, Italy).^1^

All the involved subjects were right-hand dominant. Eight out of ten could walk alone and the other two could walk with the support of a walker. The study design and protocol, including subject privacy and data treatment plan, were approved by the Ethical Committee of “Fondazione Casa Sollievo della Sofferenza” (Approved on June 12, 2017; Prot. Code H2020-738,251 ACCRA). Written informed consent to participate in the study and to use the data for research purposes was obtained from each participant. During the experimental trials, a technician and a doctor assisted the participants and thus to promptly intervened in case of necessity.

### Experimental Protocol

Before starting the experiment, the technician taught the user how to use ASTRO during the walk. Particularly, thanks to the installed “Smart Drive Mode” (see System Description Section), if the user would like to activate the robot, he had to increase the handgrip strength of both hands and start walking. If the participant wished to turn left/right, he/she had to increase the handgrip strength of the correspondent hand. As soon as the participant felt confident in using ASTRO, the test could start.

Similarly to [55], in the beginning, the participants were asked to sustain their maximum force on the dynamometer for 5 s with elbow flex at ~ 90° and then release their grasp. This procedure was repeated for both hands. After that, they were requested to do the same with the ASTRO handle for 5 s. Then, each participant was asked to wear the IMU sensors on the feet and the sternum by using the elastic bands (see Fig. 2a). After, they were requested to walk in a straight line for 10 m at their normal speed with ASTRO with the smart drive mode activated. During the walking task, data from the laser, the camera, and IMUs sensors were acquired and stored thus being off-line analyzed.

## Sensing Module: Data Extraction

### Gait Segmentation

1. IMU sensors on the feet

The acceleration and angular rate data acquired with IMUs placed on feet were offline processed by using Matlab®R2019b (The MathWorks, Inc., Natick, MA, USA). A fourth-order low-pass digital Butterworth filter was utilized with a 5 Hz cut-off frequency for eliminating high-frequency noise [56]. Custom algorithms were developed to extract motor parameters during the walking task. The gyroscope signal perpendicular to the motor direction was analyzed. For each gait cycle, the start (TS) and the end (TE) events were identified from the algorithms; then, the segmentation procedure divided the gait cycle into four phases: stance (ST), heel-off (HO), swing (SW) and heel-strike (HS). These phases were used in the algorithms to extract the gait temporal parameters as detailed in [57]. The same parameters were extracted from the left (L) and the right (R) foot. As concern data from the IMUs, the length of the stride (GSTRDL) was computed as the ratio between the distance (10 m) and the number of strides (GSTRD). At the end of the process, a total of 18 parameters were extracted, as reported in Table 2.

(2) Laser data on Robot

**Table 2:** Description, mean value and standard deviation (SD) of the gait features computed for the laser and the IMU data

Acronym	Features	Data extracted from IMU Mean Value (SD)	Data Extracted from Laser Mean Value (SD)	*p*	R
GTR	Gait Time of right foot	46.02 (9.52) s	44.22 (9.76) s	0.125	0.589^a^
GSTRDR	Number of Strides of right foot	19.90 (4.89)	19.6 (5.36)	0.960	0.908^a^
GSTRDLR	Stride Length of right foot	0.53 (0.14) m	0.39 (0.06) m	0.843	0.758^a^
GSTRDT)	Stride Time of right foot	2.32 (0.57) s	2.22 (0.67) s	0.827	0.654^a^
GSTRDTR_SD	SD of Stride Time of right foot	0.53 (0.43) s	0.41 (0.25) s	0.842	0.558^a^
GSWTR	Swing Time of right foot	0.44 (0.060) s	0.79 (0.14) s	0.335	0.210
GSWTR_SD	SD of Swing Time of right foot	0.086 (0.054) s	0.20 (0.07) s	0.839	0.641^a^
GSTTR	Stance Time of right foot	1.89 (0.54) s	1.40 (0.52) s	0.333	0.664^a^
GSTTR_SD	SD of Stance Time of right foot	0.50 (0.41) s	0.35 (0.25) s	0.782	0.419^a^
GTL	Gait Time of left foot	44.62 (10.18) s	44.22 (9.76) s	0.661	0.601^a^
GSTRDL	Number of Strides of left foot	19.50 (5.28) s	19.8 (5.51)	0.951	0.912^a^
GSTRDLL	Stride Length of left t foot	0.56 (0.19) m	0.35 (0.06) m	0.707	0.560^a^
GSTRDTL	Stride Time of left foot	2.33 (0.61) s	2.23 (0.69) s	0.796	0.777^a^
GSTRDTL_SD	SD of Stride Time of left foot	0.48 (0.40) s	0.35 (0.16) s	0.869	0.757^a^
GSWTL	Swing Time of left foot	0.42 (0.079) s	0.76 (0.18) s	0.591	0.508^a^
GSWTL_SD	SD of Swing Time of left foot	0.081 (0.052) s	0.21 (0.10) s	0.238	0.137
GSTTL	Stance Time of left foot	1.92 (0.55) s	1.46 (0.56) s	0.879	0.735^a^
GSTTL_SD	SD of Stance Time of left foot	0.45 (0.37) s	0.31 (0.14) s	0.842	0.689^a^

The results of the Mann-Withney test (*p*), the coefficient of the linear regression (R), and the mean absolute error (ɛ) are also reported

^a^The linear regression are significant (*p* < 0.05)

**Fig. 3:**
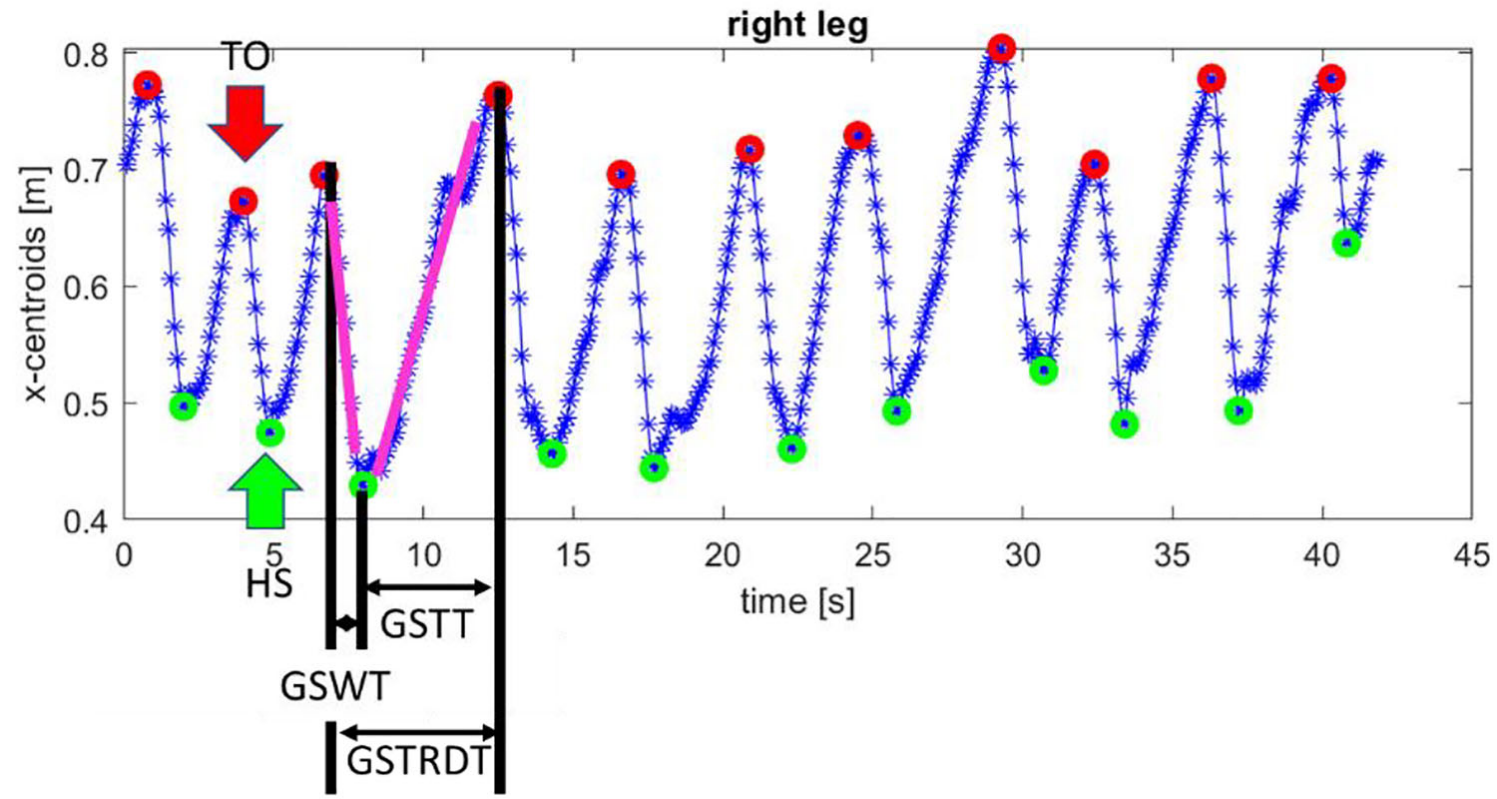
Laser data acquired during the 10 m walking test. The red points are the Toe-off (TO) instances and the red points are the Heel Strike instances. The pink lines are the length of the stride. The GSTRDT, GSWT, and GSTT are also reported

Firstly, data from the laser were filtered to remove the noise which corresponds to when the laser did not see two centroids because of several reasons (e.g. caregiver help during the walking task, camera occlusion). After, the same parameters extracted from the IMUs were computed also from the laser data for both feet. In this analysis, we considered only the data along the *x*-axis, which is the direction of the walking task. In this model, the origin of the axis (*x* = 0) corresponds to the ASTRO robot. An example of laser data is reported in Fig. 3. These signals were analyzed by computing the maximum (which corresponds to the TO) and the minimum (which corresponds to HS). By using these points, the same features were extracted. The swing phase corresponds to the segment where the distance between the robot and the legs diminishes and the stance phase corresponds to the opposite slope. It is worth noticing that, in this case, the STRDL was computed as the length of the curve between two TO points (pink lines). The segmentation of the signal is summarized in Fig. 3.

### Body Posture

1. RGBD Camera on ASTRO

Each video has been processed offline with an open-source tool named OpenPose [42]. This toolkit automatically detects 25-keypoint body pose locations and 70 fiducial facial landmarks, along with their detection confidence (0–1) from the 2D image. In our work, the camera is mounted on the upper part of the robotic platform, thus it moves accordingly to it. Since only the upper body of the user is visible in the image, only 4-keypoint body pose locations are of interest for our analysis (Fig. 2b): the left shoulder, right shoulder, neck, and nose. It is worth noticing that during the walking performance, some external users may be present in the scene. To disambiguate the main user from the external users, the body pose of the main user is updated based on his/her initial pose. Since the camera records also the depth images (along the *z*-axis), the depth information has been automatically retrieved from the pixels belonging to the detected body key points.

From these key points, several geometrical parameters were extracted. As shown in Fig. 2b, the line connecting the key points of the shoulders was computed to determine the slope (m) and the inclination (theta). These two parameters can be used to analyze the motion of the shoulders during the walk. From the RGB domain, we computed the Euclidean distance between the nose and the neck key points (DIS), computed as:

$$ DIS = \sqrt {\left( {x_{nose} - x_{neck} } \right)^{2} + \left( {y_{nose} - y_{neck} } \right)^{2} } $$

This parameter was chosen as a descriptor of the tilt motion of the torso. The tilt motion of the torso was estimated also by the variation of the position of the neck in the depth domain in terms of minimum (ZMIN) and maximum (ZMAX) values. Additionally, the root mean square error of the neck position in the depth domain (ZRMSE) was also computed to evaluate the mean oscillation during the walking task. Similarly, the Euclidean distance was used to compute the displacement of the nose (NOSE) and neck (NECK) position among consecutive frames, as:

$$ d\left( {p_{f} ,p_{f - 1} } \right) = \sqrt {\left( {x_{f} - x_{f - 1} } \right)^{2} + \left( {y_{f} - y_{f - 1} } \right)^{2} } $$

where ${p}_{f}=({x}_{f},{y}_{f})$ is the pixel location belonging to key the nose (or neck) at frame $f$. These two parameters can be descriptive of the motion of the head and of the torso during walking activity, respectively. All the parameters extracted from the camera are listed in Table 3.

2) IMU on Sternum

**Table 3:** Description, mean value, and standard deviation (SD) of the features related to the selected body joints and the handgrip strength extracted from the RGBD camera and the force sensors respectively

Acronym	Features	Source	Mean (SD)
m	Shoulder Slope	Camera	0.043 (0.075)
m_SD	SD of the Shoulder Slope	Camera	0.047(0.016)
THETA	Shoulder Inclination	Camera	2.46 (4.29)°
THETA_SD	SD of the Shoulder Inclination	Camera	2.64 (0.86) °
DIS	Nose-Neck Distance	Camera	144.64 (14.82) pixel
DIS_SD	SD of the Nose-Neck Distance	Camera	17.29 (7.28) pixel
NOSE	Nose Displacement	Camera	2.97 (0.55) pixel
NOSE_SD	SD of the Nose Displacement	Camera	2.38 (0.35) pixel
NECK	Neck Displacement	Camera	2.88 (0.43) pixel
NECK_SD	SD of the Neck Displacement	Camera	2.05 (0.27) pixel
Zmin	Minimum Neck Variation along the z-axis	Camera	575.30 (71.84) mm
Zmax	Maximum Neck Variation along the z-axis	Camera	794.50 (41.96) mm
ZRMSE	Root mean square error of the Neck along the z-axis	Camera	35.57 (11.47) mm
THETAI	Average value of the sternum angular excursion	IMU	1.08 (1.88) °
THETAI_SD	SD of the value of the sternum angular excursion	IMU	1.81 (0.79) °
MFR	Maximum Force Right Hand	Force	76.52 (39.42) N
MFL	Maximum Force left Hand	Force	78.01 (37.06) N

Angular velocity from the IMU placed on the sternum was analyzed to estimate the angular excursion of the body along the direction of the path (*z*-axis as in Fig. 2b). These data were synchronized with the ones acquired with the IMUs placed on the feet. We consider, for each step, the HS of the right foot and the HS of the left foot as the points to segment the angular velocity around the z-axis obtained from the gyroscope placed on the sternum. Then we extract three features to characterize the body oscillation: the average oscillation (THETAI) and its standard deviation (THETAI_SD). The signal was integrated to extract the angle of the body oscillation.

### Handgrip Strength

1. ASTRO handle

Similarly to [55], the outputs of the 16 force sensors mounted on the ASTRO handle (8 in correspondence to the left hand and 8 in correspondence to the right hand) were added up to obtain the two profiles of the total hand grip strength over the time. Then, the maximum force values were identified as the peak of the slope for each subject.

(2) Camry dynamometer

Camry dynamometer has a digital display where the maximum force is shown. At the end of the test, the experimenter reported manually the force into the data record.

### Data Comparison

The following comparisons were performed to answer Research Question I.

#### Comparison of Gait Analysis

At the end of this process, we had two datasets, one from the laser data and one from the IMUs data. The normal distribution of the features was verified using the Kolmogorov–Smirnov test of normality. Since all the parameters were not normally distributed, the non-parametric statistic was applied. Statistical Mann–Whitney *U* signed test was used to investigate if there are significant differences (*p* < 0.05) among the gait parameters measured with the IMUs and with the Laser. A linear regression analysis, as a linear approach to model the relationship between the dependent and independent variables, was performed to further evaluate the accuracy of the amplitude measurements considering the average values of the parameters measured from the two systems (listed in Table 2). The coefficient of linear regression (R) and its related significance p-value parameters were calculated (significant value *p* < 0.05). This comparison extends the one described in [55], which included only young subjects.

#### Comparison of Handgrip Strength

The maximum value of the handgrip strength of the dominant hand (the right for all participants) was compared with the data measured with the dynamometer. The normal distribution of the maximum forces was verified using the Kolmogorov–Smirnov test of normality. As all raw scores were not normally distributed, the non-parametric Spearman (ρ) correlation coefficient was used. Besides, to investigate similarity in the performances of the two tests, the coefficient of linear regression (R) and its related significance* p*-value parameter were calculated. The Root Means Square Error (RMSE) was also computed to estimate the goodness of the fitting.

#### Comparison of the Body Oscillation Analysis During the Walk

Correspondent features extracted with the RGBD camera compared with the ones extracted with the IMU. The selected features considered in this comparison were the ones related to the angular excursion of the torso (average value, maximum value and standard deviation) along the *z*-axis during the walk. As in the previous comparison, the data normality distribution was prior verified as previously described; since the data were not normally distributed, the non-parametric statistic was applied. The Mann–Whitney* U* signed test was used to verify whether there were differences between the THETA and THETAI, THETA_SD and THETAI_SD. Similar to the other comparisons, the coefficient R was computed between these couple of features.

## User Profiling: Data Visualization

The dataset acquired from the laser, the dataset acquired from the RGBD camera, and the dataset on the handgrip strength were then combined to answer Research Question II. Particularly, two-dimensionality feature reduction and visualization methods were applied to visualize and aggregate the data in a bidimensional space with the purpose to use these results as the output of the perception module (i.e. user profiling) and input of the decision support tool for the clinicians. Indeed, data from body shape, gait, and force were correlated with physical frailty (sarcopenia), thus if ASTRO can measure and group users that have similar behavior, it could offer visual support also for the clinician.

The selected methods were the T-distributed Stochastic Neighbor Embedding (t-SNE) [58] and the non-classic multidimensional scaling (nMDS) based on dissimilarities between points. All the analysis was performed using Matlab (2019b). t-SNE is an unsupervised and non-linear dimensionality reduction technique that focuses on keeping similar points close in the reduced features space. In this paper, we used the built-in function of Matlab *tsne* selecting the ‘*exact*’ algorithm and the ‘*euclidean*’ methods for calculating the distance between two points. As for the nMDS, we used the *midscale* Matlab function with Sammon’s non-linear mapping criterion for the metric scale. In the latter case, the stress parameter was used to evaluate the goodness of the reduction process.

## Results

This paper aims to investigate two research questions, that were focused on the implementation of the sensing and the perception modules of ASTRO robot. ASTRO robot was satisfactorily tested in real environments with real patients. All the participants completed the 10-m walk task, including the two people with limited walking abilities.

The data from the laser, the camera, and the force sensors were off-line processed to extract all the parameters related to the gait, the body posture, and the handgrip strength (RQI). Particularly, at the end of the features extraction process described in the previous section, 18 features were extracted from the laser (i.e. 9 for each foot), 13 features were extracted from the RGBD camera to describe the body orientation during the walking task and 2 features were extracted from the handgrip strength. A total of 33 parameters described and characterized the patients. The mean value and standard deviation computed for the 10 participants in the experimentation are reported in Table 2 (features extracted from gait) and in Table 3 (features extracted from RGBD camera and the force sensors).

As concern the comparison of the gait analysis, the Mann–Whitney U signed test was computed between the correspondent features extracted from the IMUs and the ones extracted from the laser. The achieved results reveal that all the extracted parameters were not significantly different (*p* > 0.05) which means that the two vectors of features extracted from the two systems (i.e. laser and IMUs) come from the same population. Additionally, except for the GSWTR and GSWT_SD, all R coefficients are significant (*p* < 0.05); it is worth underlining that, the R for GSTRD is higher than 0.95 for both feet. The complete results are depicted in Table 2.

As for the comparison of the handgrip strength, the average dynamic force profile was computed as the sum of each force sensor to extract the maximum force value for the dominant hand. The correlation between the maximum values of the handgrip forces underlines a good and significant (*p* < 0.001) correlation of the hand force measured with the dynamometer. Indeed, the ρ is equal to 0.84, and the regression analysis confirms a good linear dependency of the two measures resulting in a high value of R and low RMSE (0.74 and 5.12 N respectively).

The comparison of the body oscillation during the walk confirms a not significant difference (*p* < 0.05) for the measurement of the average angular excursion of the torso with the two systems. Whereas there are significant differences (*p* < 0.05) in the standard deviation. There were not any significant linear regression coefficients. The average values are reported in Table 3.

**Fig. 4:**
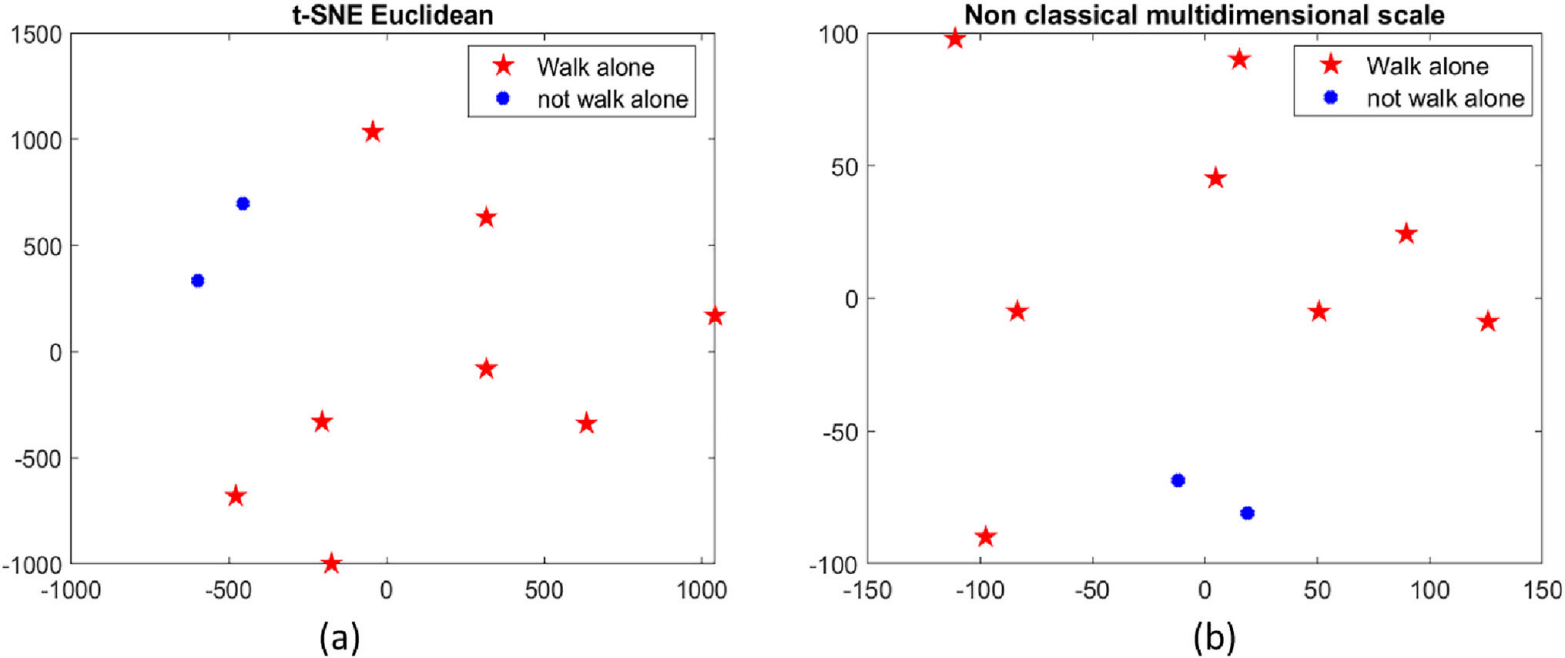
**a** Data visualization with the t-SNE method; **b** Data visualization with the non-classical multidimensional scale

As for research question II, the 9 features that describe the gait of the right foot (average values and SD), the features related to the body slope (13 features), and the maximum values of the handgrip strength computed with ASTRO handle were combined in a single dataset (for a total of 24 features), and visualized with the t-SNE and the nMDS methods. Figure 4a and b depict the new distribution of the features in the 2D spaces with the t-SNE and the nMDS approaches respectively. The stress index value equal to 0.95% obtained for the nMDS method underline a good feature reduction process for the proposed dataset. The goal of these unsupervised methods was to explore the relationship among instances, giving an overview of how each instance is represented in the feature space. Indeed, they preserve the similarity among instances in the bi-dimensional visualizations. Similar instances were then visualized closer than others. In our cohort, we had two participants that are not able to walk without external aid (the blue dots) whereas the remaining participants (red stars) can walk alone, the operative hypothesis was that they could be similar instances in the feature space. From the visual inspection, it is evident that these approaches potentially can group the instances of the dataset according to the similarities among points.

## Discussion

This paper presented a robot model that uses information on the user profile as input for the *decision-making module* (Fig. 1). That information can follow two paths: (i) it can be used from the decision support tool for supporting the clinician in supervising the status of the older persons and modify (if necessary) the information on the user stored in the model, and (ii) it can be used from the interaction module to modulate the behavior of the robot during the interaction. Additionally, the work presented a feasibility study where the data related to the sarcopenia during a 10-m walking task were combined at glance to provide the clinician feedback on the performance.

The rationale behind this approach is that the same feature can provide different information related to the interaction/engagement with the robot and the clinical status. For instance, in this paper, the handgrip strength is used as input for the perception module, and it is also used by ASTRO to adapt the angular and linear velocity in real-time during the walking task. The formulated RQI aimed to evaluate the multi-modal sensing modules, by evaluating the accuracy of the extracted features and proposing a comparison with the gold standard model. These tests were fundamental steps to be verified before using ASTRO in real/clinical applications. The results underline that laser sensor could be used to measure comparable features extracted from the IMUs sensors, widely used in similar applications. Remarkably, also the measurement of the dominant handgrip strength is highly correlated to the one measured with the dynamometer. These results are aligned with the ones we obtained involving young and healthy subjects [55]. These feasibility results also suggest that the camera on the back could be an added value to measure the body shape of the subject during the walking task providing interesting parameters to be shown to the doctors/formal caregivers. Indeed, the angular excursion calculated with the IMU placed on the sternum is comparable to the one extracted from the camera.

RQII is focused on the perception module that aggregates data at the features level, proposing two methods to visualize the output in a bidimensional space using non-classical and unsupervised techniques. From a visual inspection, the obtained results (Fig. 4) underline that the extracted features set has the potential to characterize the user’s profile in terms of their residual walking abilities. According to the results, ASTRO can be suitable to monitor the performance of a frail user because it can measure and combine different features (i.e. walking, posture, hand strength) that can accurately characterize the motor status of frail people without the need to equip them with additional devices (e.g., wearable sensors, smartphone). It is also remarkable that the proposed visualization techniques can cluster the subjects according to the residual motor abilities using the parameters extracted from the contactless ASTRO sensors. Indeed, the two subjects that were not able to work alone are close in the figures. Clinicians can use this information as a decision support tool to monitor/assess the performance of the users and see if problems occur. Those techniques are based on algorithms that will enhance similarities among points, similarities that, the proposed robotic multi-modal sensor module can estimate through the selected feature set.

For those people who will use ASTRO, the caregiver could also monitor the walking parameters during the mobility service. From a clinical perspective, indeed, gait abnormalities are correlated with several cognitive and physical impairments such as Parkinson's disease, sarcopenia, Mild Cognitive Impairments (see Table 1), thus ASTRO could provide insights for the clinicians in detecting and monitoring a pathology over the time. The assessment of the gait performance over time is important, and, in this context, the ASTRO robot has the potential to do it in a “transparent” way for the user, avoiding asking to wear specific sensors.

The paper presents the feasibility study on sarcopenia-related features as an example since other features can be extracted and used to profile the user and to orchestrate the HRI. Similarly, data from the camera could be also analyzed to extract information related to the facial expression to investigate engagement during the interaction. Remarkably, the sensing abilities of the robotic platform will guide the performances of the models, thus the robot must integrate different types of sensors according to the service it will be involved in. For future exploitation of such an approach, the robot should be able to select and use the appropriate multi-modal information thus to profile the user correctly and guide the system in planning the next actions. In this sense, the AI can predict the future state according to the internal model and the user profile modulating the inputs for the decision support module. Indeed, it is important to tailor the action by modulating the “what” and “how” of the interaction. In other words, the content of the interaction and the modality of the interaction should be planned.

It is evident that proposing a model with the human-in-the-loop guarantees a high level of system adaptability and customization [3]. Indeed, in this feasibility study, we include only the information coming from the sensors to produce the output of the perception module. In future applications, the system could fuse, at a different level, that information with others coming from the context (and/or inserted by the user) that can provide information on the gender, the cultural background, the user personality such as on the cognitive and physical profile (Fig. 1). Indeed, the HRI is highly dependent on all these issues, so it is important to include them in the loop. Additionally, the human-in-the-loop also receives the output of the decision support module, so it can validate in real-time if the output of the perception module is aligned with the user’s profile. Indeed, it can send feedback on the accuracy of the prevision. The system can potentially use this feedback to manage unseen situations and meliorate the accuracy of the AI algorithms.

This paper presents some limitations related to the number of people involved in the study and the a-synchronous process of user profiling. Immediate future studies should be planned to enlarge the dataset creating a cohort of participants that have different physical and cognitive impairments thus improving the accuracy of user profiling quantitatively. Additionally, the developed modules to extract the features will be implemented and tested in real-time thus testing the interactive modules. Other studies could be planned to acquire and analyze other social cues (e.g. facial expression, hand and body gestures, gaze) that could be linked with other clinical aspects.

## Conclusion

The aim of this paper was twofold. Firstly, it aims to present and discuss the presented behavioral model (Fig. 1), then it presents the feasibility test to implement the perception module. The key contribution of this paper is to present the dual use of the output of the perception module. Indeed, we used the handgrip strength to modulate the walking support with the AI techniques to adapt ASTRO during the walking test [20] and to profile the user. Additionally, the paper preliminary test in a real setting with older adults the sensing and the perception modules such as the ability to profile the users using a multimodal approach.

In this context, two research questions were outlined and investigated. The presented analysis confirms a positive result for all the formulated RQs since the features extracted from data collected with ASTRO are comparable with the ones extracted with traditional devices. It is also worth mentioning that the user’s profile obtained from the data is aligned with the participant’s residual walking abilities. Indeed, people that are not able to walk alone are close points in the bidimensional visualization (Fig. 4). This result suggests that all the data can be aggregated in a meaningful way and visualized “at a glance” to be used by the clinician to monitor the status of the users and its progression over time Additionally, the outcome for the profile can be used also by ASTRO to tailor and adapt the physical interaction during the walking task. This paper presents a feasibility study on features related to Sarcopenia, but researchers could use this approach to investigate other features connected with other HRI and clinical outcomes.

## Footnotes

1 Hospital “Casa Sollievo della Sofferenza” official website: https://www.operapadrepio.it/it/ospedale.html.
